# Supplementary material for: Increasing Frequency of G275E Mutation in the Nicotinic Acetylcholine Receptor α6 Subunit Conferring Spinetoram Resistance in Invading Populations of Western Flower Thrips in China
Source: Insects. 2022 Mar 28;13(4):331. doi: 10.3390/insects13040331 (PMC9029678; doi:10.3390/insects13040331)
Supplement: Supplementary file 1 [file insects-13-00331-s001.zip › insects-1636733-supplementary.pdf]

Table S1 Collection information for *Frankliniella occidentalis* populations

Period	Year	Population	*n*	Location of collecting site	Longitude (°E)	Latitude (°N)	Host
1st stage	2010	USDZ	23	State of Texas, USA	-99.9018	31.9686	-
	2010	USJZ	17	State of California, USA	-119.4179	36.7783	-
	2009	XJKL	17	Urumqi, Xinjiang Uygur Autonomous Region	87.2313	43.3899	Cucumber
	2009	LNCY	31	Liaoning Province,Chaoyang	120.4504	41.5737	Hollyhock
	2009	BJHD	19	Haidian District, Beijing	116.2872	39.9439	Spinach
	2009	BJFS	36	Hancunhe, Fangshan District, Beijing	115.9643	39.6020	Cucumbe
	2010	BJYL	30	Lvfulong, Yanqing District, Beijing	115.9750	40.4567	Cucumber
	2010	BJMT	62	Mentougou District, Beijing	116.1017	39.9403	Pepper
	2010	SDQD	10	Qingdao City, Shandong Province	120.3826	36.0671	Cucumber
	2010	JSYZ	37	Yangzhou City, Jiangsu Province	119.4130	32.3942	Towel gourd
	2010	XZLS	10	Tibet Province, Lasa	91.1409	29.6456	Cucumber
	2010	GZGY	40	Guiyang City, Guizhou Province	106.6302	26.6477	Chinese rose
	2010	YNKC	23	Chenggong District, Kunming City, Yunnan Province	102.8211	24.8869	-
	2010	YNKJ	29	Honghe Prefecture, Yunnan Province	102.6024	24.6761	-
	2010	YNHH	20	Honghe Prefecture, Yunnan Province	100.5874	25.2094	Corn
	2011	BJHD	22	Haidian District, Beijing	116.2929	39.9497	Bean
	2011	BJDX	22	Daxing District, Beijing	116.3486	39.7326	Pepper
	2013	BJHD	22	Haidian District, Beijing	116.2929	39.9497	-
2nd stage	2014	BJYL	22	Lvfulong, Yanqing District, Beijing	116.0749	40.5520	-
	2014	BJDJ	21	Qingyundian Town, Daxing District, Beijing	116.5543	39.6740	Cucumber
	2015	SXBJ	24	Baoji City, Shaanxi Province	107.2138	34.3499	Canna
	2016	BJYL	22	Lvfulong, Yanqing District, Beijing	116.0749	40.5520	Eggplant
	2016	BJMY	22	Miyun District, Beijing	116.8267	40.3272	-
	2016	BJTZ	22	Tongzhou District, Beijing	116.7723	39.7563	Pepper
	2016	BJDQ	22	Yufa Town, Daxing District, Beijing	116.3221	39.5168	Eggplant
	2016	GSZY	21	Zhangye City, Gansu Province	100.4563	38.9314	Morning glory
	2016	NXYC	22	Yinchuan City, Ningxia Hui Autonomous Region	106.2742	38.4817	Canna
	2017	BJYL	21	Lvfulong, Yanqing District, Beijing	116.0749	40.5520	Cucumber
	2017	BJMY	22	Miyun District, Beijing	116.8267	40.3272	Pepper
	2017	BJCH	22	Xiaotangshan, Changping District, Beijing	116.4060	40.1845	Cucumber
	2017	BJCQ	22	Xiaotangshan, Changping District, Beijing	116.4562	40.1795	Eggplant

	2017	BJSY	22	Shunyi District, Beijing	116.8830	40.1478	Eggplant
	2017	BJPG	22	Pinggu District, Beijing	117.1274	39.9042	Pepper
	2017	BJHD	22	Haidian District, Beijing	116.2929	39.9497	Eggplant
	2017	BJFS	22	Hancunhe, Fangshan District, Beijing	115.9643	39.6020	Pepper
	2017	*BJDJ	22	Qingyundian Town, Daxing District, Beijing	116.5543	39.6740	Pepper
	2017	BJDQ	22	Yufa Town, Daxing District, Beijing	116.3221	39.5168	-
	2017	SDSG	21	Shouguang City, Shandong Province	118.9458	36.8857	Pepper
	2017	HBXN	22	Xianning City, Hubei Province	114.3285	29.8471	Bitter gourd
	2018	BJHD	22	Haidian District, Beijing	116.2929	39.9497	Eggplant
	2018	BJCY	22	Chaoyang District, Beijing	116.4496	39.9264	Cucumber
	2018	BJDQ	20	Yufa Town, Daxing District, Beijing	116.3221	39.5168	Strawberry
	2018	YNDL	22	Dali City, Yunnan Province	100.5874	25.2094	Bean
3rd stage	2020	NMHS	21	Saihan District, Hohhot, Inner Mongolia	111.7993	40.7194	Pepper
	2020	NMHH	19	Helinger County, Hohhot City, Inner Mongolia	111.7111	40.4943	Bitter gourd
	2020	BJYL	22	Lvfulong, Yanqing District, Beijing	116.0749	40.5520	Cucumber
	2020	BJYB	22	North Vegetable Garden, Yanqing District, Beijing	115.9351	40.4274	Cucumber
	2020	BJYG	24	Guangjitun, Yanqing District, Beijing	116.0158	40.4893	Pepper
	2020	BJYD	24	Donglongwan, Yanqing District, Beijing	115.9978	40.4694	Pepper
	2020	*BJPG	87	Pinggu District, Beijing	117.1274	39.9042	Loofah
	2020	BJHD	22	Haidian District, Beijing	116.2940	39.9502	Loofah
	2020	BJFS	22	Hancunhe, Fangshan District, Beijing	115.9604	39.6079	Loofah
	2020	*BJDQ	22	Yufa Town, Daxing District, Beijing	116.3221	39.5168	Pepper
	2020	*BJDJ	22	Qingyundian Town, Daxing District, Beijing	116.5543	39.6740	Pepper
	2020	*BJTZ	22	Tongzhou District, Beijing	116.5672	39.7553	Pepper
	2020	SCDY	19	Deyang City, Sichuan Province	104.4140	31.0051	Loofah
	2020	SCCD	22	Chengdu, Sichuan Province	104.2148	30.7855	Pepper
	2020	YNYX	22	Yuxi City, Yunnan Province	102.5536	24.3577	-
	2021	BJYZ	21	Zhuojia Village, Yanqing District, Beijing	115.9948	40.5141	Strawberry
	2021	*BJHD	30	Haidian District, Beijing	116.2929	39.9497	Chinese rose
	2021	*BJDS	27	Sijiyangkun, Daxing District, Beijing	116.3039	39.6066	Pepper
	2021	HNZZ	22	Zhengzhou City, Henan Province	113.6314	34.7534	Pepper

n, number of individuals examined in each population; *, population used in bioassay; -, data is missing.
